# Temperature dependence of the rigid amorphous fraction of poly(butylene succinate)

**DOI:** 10.1039/d1ra03775g

**Published:** 2021-07-27

**Authors:** Maria Cristina Righetti, Maria Laura Di Lorenzo, Patrizia Cinelli, Massimo Gazzano

**Affiliations:** CNR-IPCF, National Research Council – Institute for Chemical and Physical Processes Via Moruzzi 1 56124 Pisa Italy cristina.righetti@pi.ipcf.cnr.it; CNR-IPCB, National Research Council – Institute of Polymers, Composites and Biomaterials Via Campi Flegrei 24 80078 Pozzuoli Italy; University of Pisa, Department of Civil and Industrial Engineering Largo Lazzarino 2 56122 Pisa Italy; CNR-ISOF, National Research Council – Institute of Organic Synthesis and Photoreactivity Via Gobetti 101 40129 Bologna Italy

## Abstract

In this contribution the temperature evolution of the constrained or rigid amorphous fraction (RAF) of biodegradable and biocompatible poly(butylene succinate) (PBS) was quantified, after detailed thermodynamic characterization by differential scanning calorimetry and X-ray diffraction analysis. At the glass transition temperature, around −40 °C, the rigid amorphous fraction in PBS is about 0.25. It decreases with increasing temperature and becomes zero in proximity of 25 °C. Thus, at room temperature and at the human body temperature, all the amorphous fraction is mobile. This information is important for the development of PBS products for various applications, including biomedical applications, since physical properties of the rigid amorphous fraction, for example mechanical and permeability properties, are different from those of the mobile amorphous fraction.

## Abstract 2

At room temperature and at the human body temperature, all the amorphous fraction is mobile in poly(butylene succinate).

## Graphical abstract

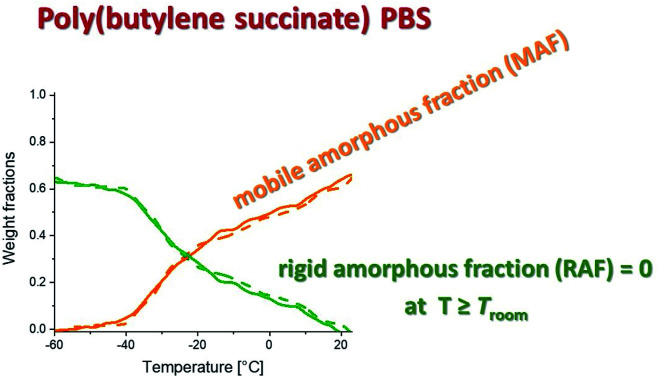

## Introduction

Poly(butylene succinate) (PBS) is a biodegradable and biocompatible polyester produced by polycondensation of succinic acid and 1,4-butanediol. Both monomers can be petrol-derived, but can be produced also from renewable resources: succinic acid *via* fermentation of carbohydrates,^1,2^ and 1,4-butanediol through hydrogenation and reduction of succinic acid.^3^ Thus, considering the growing tendency towards production of sustainable materials, PBS appears a very interesting polymer, as attested by an increasing amount of papers devoted to the properties and utilization of this polyester.

The low glass transition temperature of PBS, well below room temperature, and the relatively high melting temperature (above 100 °C) are similar to those of most common polyolefins. Also the mechanical properties do not differ substantially from those of polypropylene, or low- and high-density polyethylene.^4^ Currently PBS is commercialized mainly as a component for compostable bags, mulching films, nets, nonwoven sheets, beverage cups and food utensils,^5^ whereas PBS-based composites are of interest for food packaging.^5–7^ PBS is used in biomedicine, to make bone marrow stem cells, in tissue repair and engineering, to fabricate scaffolds that enhance the regeneration of bone in the dental socket, and also blended with chitosan for antimicrobial and antitumor activity, improved protein absorption and rapid cell growth.^4,8–14^ In addition, due to its high flexibility and toughness, PBS has been widely used in blends with other biodegradable and/or bio-based polymers, such as poly(lactic acid), poly(hydroxybutyrate), poly(propylene carbonate).^15–19^ Although these blends are generally immiscible, materials with improved ductility and reduced brittleness can be obtained.

PBS is a semi-crystalline polymer, characterized by a quite high crystallinity degree (35–45%). Upon melt crystallization, α-crystals grow, whereas upon stretching, a different crystal modification, β-form, is obtained reversibly by solid–solid transition.^20,21^ Both phases are characterized by a monoclinic cell containing two repeating units, arranged with chain conformation TTTGTḠTTTT in the α-phase and with all chains in *trans* conformation in the β-form. PBS crystallization rate is high: the processes is completed in about 1 min at −10 °C, and in less than 10 s in the temperature range between 10 and 80 °C.^22^ This means that quenched PBS cannot be maintained in the amorphous state at room temperature.

In polymers, a semi-crystalline structure necessarily implies the presence of a constrained interphase at the amorphous/crystal boundary, due to the covalent bonds that connect the crystalline and amorphous regions. The nano-metric constrained amorphous interphase is generally called rigid amorphous fraction (RAF), because it is characterized by reduced chain mobility compared to the mobile amorphous fraction (MAF).^23^ The MAF vitrifies/devitrifies at *T*_g_, whereas vitrification/devitrification of progressively more constrained amorphous regions, *i.e.* RAF, occurs at progressively higher temperatures, as clearly reported in quite recent years.^23,24^ RAF percentages of about 20–30 wt% have been determined for several polymers at *T*_g_, whereas its temperature dependence has been determined only for few polymers.^24^ Also for PBS, RAF amount of this order of magnitude has been measured at *T*_g_.^25–27^ Recent studies have demonstrated that the rigid amorphous fraction influences the performance of semi-crystalline polymers, because many physical properties of the RAF are different from those of the crystalline and the mobile amorphous fractions.^28–35^ Experimental evidences and theoretical modelling have demonstrated that the elastic modulus of the RAF (*E*_RA_) is between those of the crystalline (*E*_C_) and mobile amorphous (*E*_MA_) fractions, in the order *E*_MA_*< E*_RA_ < *E*_C_.^29,31,32^ On the other hand, the density of the RAF (*ρ*_RA_) is lower than that of the MAF (*ρ*_MA_), due to the higher RAF vitrification temperature,^32,33^ so that the order of the densities turns out to be *ρ*_RA_ <*ρ*_MA_ <*ρ*_C_, where *ρ*_C_ is the density of the crystalline phase.

A rigid amorphous fraction is present in almost all semi-crystalline polymers,^23^ and can exist in nano-layered polymers,^36–38^ block copolymers,^39,40^ and polymer nanocomposites.^41–49^ Since it can constitute a large fraction of the overall material, it has large impact on properties. Huge research efforts have been devoted in the latest years to a thorough understanding of the RAF, as recently reported in a review,^24^ where the experimental methods used to monitor the RAF, the influence of thermal history and crystal structure/morphology on the RAF, and the RAF influence on material properties are summarized.

These considerations imply that quantitative information on the RAF is essential to design industrial processes for specific applications of semi-crystalline polymers. If mechanical and barrier properties have to be fine-tuned, for example in case of films for food packaging, it needs to be taken into account that the rigid amorphous and crystalline fractions have opposite effects on barrier properties,^34,35^ whereas they together contribute to the material stiffness.^28–32^ Thus, a proper balance between crystalline and rigid amorphous fractions is essential to develop a material with specific gas/vapor permeability and flexibility.

Needless to say, the above considerations on the RAF density and modulus refer to vitrified RAF, since, once the RAF is mobilized, its properties become similar to the MAF. This clarifies the importance of knowledge on the RAF glass transition. Unfortunately literature information on RAF devitrification are available only for a few semi-crystalline polymers, including among others poly(l-lactic acid), poly(1-butene), poly[(*R*)-3-hydroxybutyrate], poly(ethylene terephthalate), poly(butylene terephthalate),^50–54^ but to date not yet disclosed for PBS. Also the physical properties of the PBS rigid amorphous fraction (for example mechanical or permeability properties) have not yet been investigated.

With this manuscript, data on devitrification of the RAF in PBS are presented, with the aim to favor the interpretation and prediction of physical properties, in particular mechanical and barrier properties, of PBS-based materials. For an accurate quantification of the RAF evolution as a function of the temperature, a preliminary thermodynamic characterization of PBS was performed by differential scanning calorimetry and X-ray diffraction analysis.

## Experimental

### Chemicals

Additive-free poly(butylene succinate) (PBS) was kindly supplied by SIPOL (Mortara, Italy). The number-average molar mass (*M*_n_) and the weight-average molar mass (*M*_w_) are 27.8 × 10^3^ and 72.7 × 10^3^ g mol^−1^, respectively, as determined by gel-permeation chromatography (GPC). The as received PBS chips were dried under vacuum overnight at 60 °C, then compression-moulded into 200 μm thick films. Compression-moulding was performed with a Collin Hydraulic Laboratory Forming Press P 200 E at 130 °C for 3 min, then the films were cooled to room temperature by cold water circulating into the press plates. GPC analyses revealed no sizable change of molar mass of PBS after compression-moulding.

### Thermal analysis

Differential Scanning Calorimetry (DSC) measurements were performed with a Perkin Elmer Calorimeter DSC 8500 equipped with an IntraCooler III as refrigerating system. The instrument was calibrated in temperature with high purity standards (indium, naphthalene, cyclohexane) according to the procedure for standard DSC.^55^ Enthalpy calibration was performed with indium. Dry nitrogen was used as purge gas at a rate of 20 mL min^−1^. To gain precise heat capacity data from the heat flow rate measurements, each scan was accompanied by a blank run with an empty pan. The sample mass was lower than 10 mg, whereas the mass of the blank and sample aluminium pans matched within 0.02 mg. The temperature of the samples upon heating was corrected for the thermal lag, determined as average by using different standard materials. This lag was 0.05 min, which, for the heating rates of 2 and 10 K min^−1^, corresponds to a temperature correction of −0.1 and −0.5 K respectively, whereas for the cooling rates of 2, 5, 10 and 20 K min^−1^ to corrections of +0.1, +0.25, +0.5 and +1.0 K, respectively.

To measure the thermodynamic solid and liquid specific heat capacities (*c*_p,s_ and *c*_p,l_), PBS samples were heated to 150 °C, and maintained at this temperature for 3 min, in order to erase the previous thermal history.^56^ Then these samples were quickly removed from the DSC apparatus, quenched into liquid nitrogen, and rapidly transferred to the DSC cell maintained at −70 °C. The PBS quenched samples were analysed (i) by conventional DSC from −70 °C to 150 °C at the heating rate of 10 K min^−1^, to obtain apparent specific heat capacity (*c*_p,app_) curves, and (ii) by TMDSC, with a saw-tooth modulation temperature program, at the average heating rate of 2 K min^−1^, with a temperature amplitude (*A*_T_) of 0.5 K and a modulation period (*p*) of 120 s, to obtain average specific heat capacity (*c*_p,ave_) curve and reversing specific heat capacity (*c*_p,rev_) curve. According to the mathematical treatment of TMDSC data, the modulated heat flow rate curve can be approximated to discrete Fourier series, and separated into average and periodic components.^57,58^ The average component is equivalent to the conventional heat flow rate signal under linear temperature program. Thus, the *c*_p,ave_ curve, calculated from the average heat flow rate at the average heating rate of 2 K min^−1^, corresponds to *c*_p,app_ upon linear heating rate of 2 K min^−1^. Conversely, from the periodic component, the *c*_p,rev_ curve was obtained, according to the following equation:1
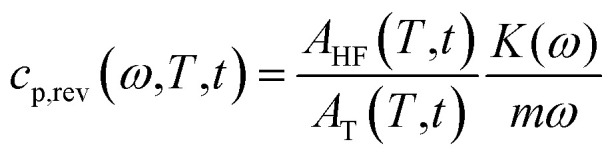
where *A*_HF_ and *A*_T_ are the amplitudes of the first harmonic of the modulated heat flow and temperature, *ω* is the fundamental frequency of temperature modulation (*ω* = 2π/*p*), *m* the mass of the sample and *K*(*ω*) the frequency-dependent calibration factor. The average *K*(*ω*) values, determined by calibration with sapphire, was 1.06 ± 0.02 and 1.00 ± 0.02 for *p* = 60 and 120 s, respectively.

Non-isothermal crystallization of PBS was performed by cooling at the rate of 2, 5, 10, and 20 min^−1^ down to 25 °C, after fusion for 3 min at 150 °C. Subsequently the samples were cooled quickly to −70 °C, and reheated at 10 K min^−1^ up to 150 °C. After non-isothermal crystallization at 10 K min^−1^, also TMDSC runs were performed with a saw-tooth modulation temperature program, at the average heating rate of 2 K min^−1^, with *A*_T_ = 0.5 K and *p* = 60 and 120 s, to obtain *c*_p,ave_ and *c*_p,rev_ curves.

### X-ray diffraction (XRD) analysis

X-ray diffraction investigation was performed at room temperature (*T*_room_) in reflection mode on PBS samples non-isothermally crystallized at 2, 5, 10 and 20 K min^−1^, by using a PANalytical X'PertPro diffractometer (Cu Kα radiation, *λ* = 0.15418 nm; X'Celerator detector). The crystal fraction (*X*_C_) was calculated from the ratio *A*_c_/*A*_tot_, where *A*_c_ is the integrated area of the crystalline diffraction and *A*_tot_ is the integrated total scattering subtracted by the incoherent scattering. For this purpose, a scan without sample and properly scaled was used for each pattern.

In addition, a heating stage Anton Paar TTK450 allowed *in situ* measurements of PBS samples crystallized at 2 and 5 K min^−1^, with temperature control of 0.1 K. The samples were heated from *T*_room_ at 15 K min^−1^. At 60, 95 and 105 °C, XRD scans were recorded with acquisition time of 90 s. During the scan collection the temperature ramp was stopped. The average heating rate was about 10 K min^−1^.

## Results and discussion

**Fig. 1:**
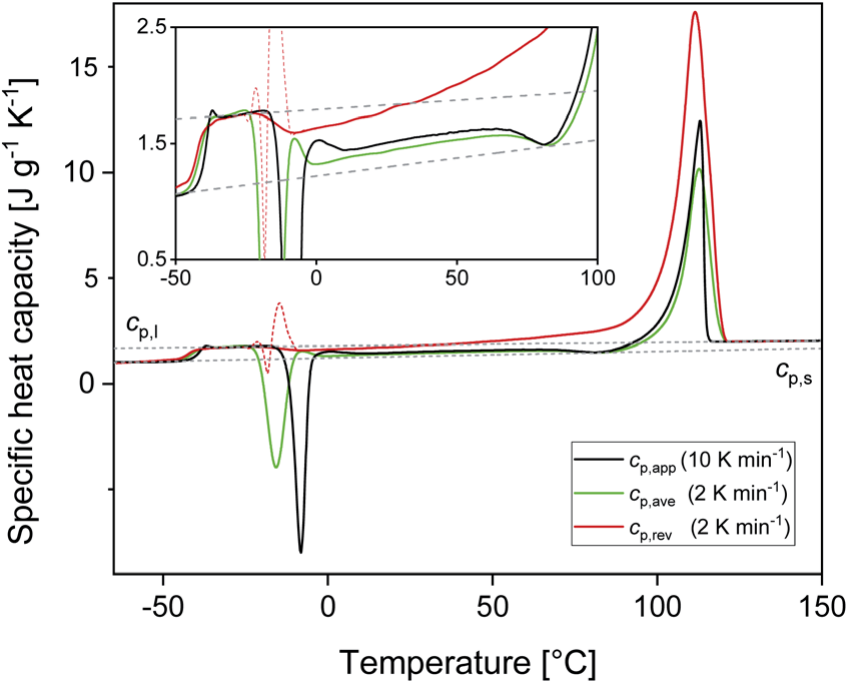
Specific heat capacities of PBS after quench from the melt as a function of temperature: apparent specific heat capacity (*c*_p,app_) at 10 K min^−1^, average specific heat capacity (*c*_p,ave_) and reversing specific heat capacity (*c*_p,rev_) at 2 K min^−1^ (*p* = 120 s, *A*_T_ = 0.5 K). The dotted lines are the thermodynamic solid and liquid specific heat capacities (*c*_p,s_ and *c*_p,l_) of PBS. The inset is an enlargement in the exothermic events region.

Fig. 1 shows the apparent specific heat capacities (*c*_p,app_) of initially amorphous PBS upon heating at 10 K min^−1^, the average specific heat capacity (*c*_p,ave_) and the reversing specific heat capacity (*c*_p,rev_) at 2 K min^−1^, together with the thermodynamic solid and liquid specific heat capacities (*c*_p,s_ and *c*_p,l_) lines, constructed by extrapolating the *c*_p,app_ and *c*_p,rev_ data from below the glass transition, and by connecting the region above *T*_g_ to the melt, respectively. The derived *c*_p,s_ and *c*_p,l_ expressions are: *c*_p,s_ = 1.22 + 0.0031*T* and *c*_p,l_ = 1.79 + 0.0016*T*, with *c*_p,s_ and *c*_p,l_ in J g^−1^ K^−1^ and *T* in °C. The *T*_g_ value, determined at half of the *c*_p,app_ increment, is −40 °C at 10 K min^−1^, and −42 °C at 2 K min^−1^. At *T*_g_, the specific heat capacity increment (Δ*c*_p,a_) is 0.63 J g^−1^ K^−1^. According to Wunderlich's “bead theory”,^59^ the bead number of the PBS repeating unit is 8, which means that each bead contributes to Δ*c*_p,a_ with 13.5 J mol^−1^ K^−1^. This value is in excellent agreement with the bead contribution reported for the homologue poly(trimethylene succinate) (13.3 J mol^−1^ K^−1^).^59^

At temperatures higher than *T*_g_, the amorphous PBS sample undergoes an intense cold crystallization at temperatures increasing with the heating rate. In parallel with the cold crystallization, the *c*_p,rev_ curve exhibits an irregular oscillation (red dashed line in Fig. 1), which is an artefact, often occurring in TMDSC analyses upon fast and intense release of latent heat.^60^ The expected downward step of the *c*_p,rev_ curve^61^ (red line in Fig. 1) has been reconstructed by interpolation. At higher temperatures, additional exothermic events occur, most likely connected to crystalline reorganization. The progressive increase in *c*_p,rev_ observed in conjunction with the exothermic processes, attests that these events follow the temperature modulation, in the sense that both crystallization and fusion take place in the two different semi-periods, respectively. The intense melting peak of the *c*_p,app_ and *c*_p,ave_ curves is centred at 113 °C. In the final melting region, *c*_p,rev_ values higher than *c*_p,app_ and *c*_p,ave_ indicate that recrystallization occurs extensively up to complete fusion.

**Fig. 2:**
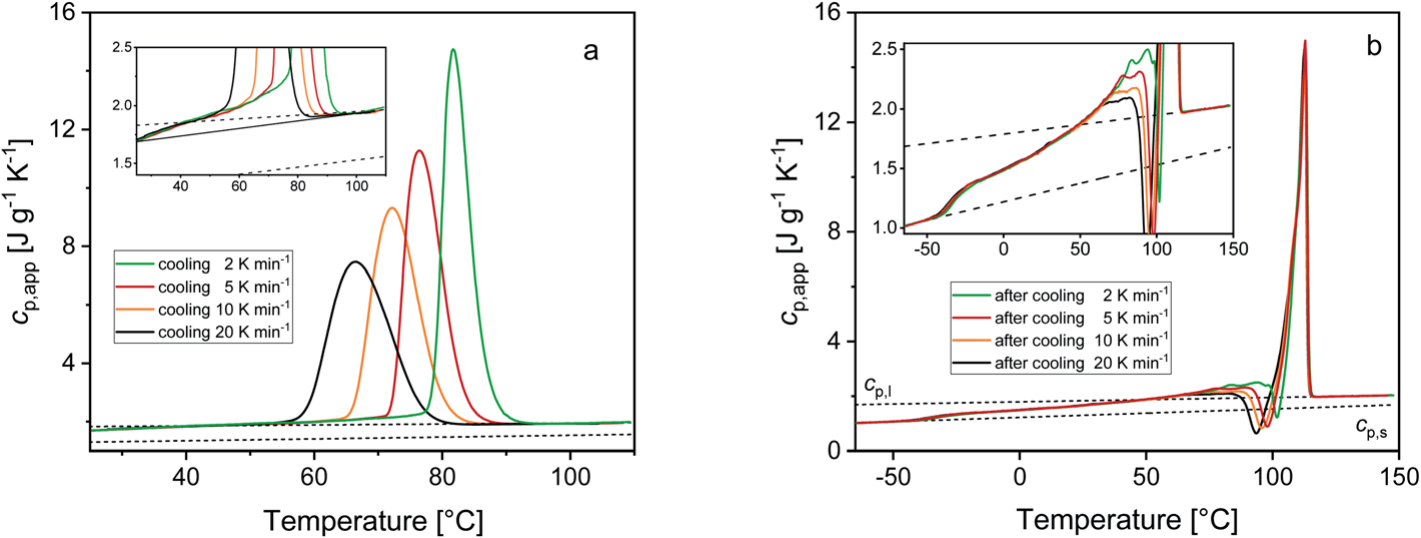
Apparent specific heat capacity (*c*_p,app_) of PBS upon cooling at different rates (a) and upon subsequent heating at 10 K min^−1^ (b). The dotted lines are the thermodynamic solid and liquid specific heat capacities (*c*_p,s_ and *c*_p,l_) of PBS. The insets are enlargements of the *c*_p,app_ curves. The thin black solid line in the inset of graph (a) is the baseline of the crystallization process.

Fig. 2 shows the *c*_p,app_ curves of PBS samples crystallized upon cooling at different rates and the *c*_p,app_ curves upon subsequent heating at 10 K min^−1^. As expected, non-isothermal crystallization (Fig. 2a) shifts to lower temperatures with increasing the cooling rate. The inset of Fig. 2a suggests that crystallization could take place down to 25 °C, although in reduced percentage with respect to the peak (less than 5%). Table 1 lists the peak temperatures of the non-isothermal crystallization process (*T*_c_), and the measured enthalpy of crystallization (Δ*h*_c_) values, with absolute value progressively decreasing with increasing the heating rate. The *c*_p,app_ curves upon heating after cooling at different rates (Fig. 2b) exhibit an approximate constant increment at *T*_g_, which means that the solid fraction (crystalline + rigid amorphous fractions) at *T*_g_ is independent of the cooling rate. The presence of endothermic and exothermic peaks in the *c*_p,app_ curves in the temperature range 55–105 °C proves that all the PBS samples undergo significant reorganization/recrystallization processes before final melting, in agreement with literature data.^22,62^ The shape of the *c*_p,app_ curves after cooling at the various rates is totally reproducible.

**Table 1:** Peak temperatures of non-isothermal crystallization (*T*_c_), enthalpy of crystallization measured by DSC, (Δ*h*_c_), crystalline fraction measured by XRD (*X*_C_), enthalpy of crystallization of 100% crystalline PBS at 
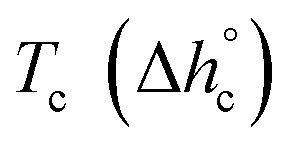
, mobile amorphous weight fraction at *T*_g_(*w*_MA_) and rigid amorphous weight fraction a *T*_g_(*w*_RA_) for PBS samples non-isothermally crystallized at different rates (estimated errors: ±0.2 °C for *T*_c_, ±0.4 J g^−1^ for Δ*h*_c_, ±0.02 for *X*_C_, ±10 J g^−1^ for 
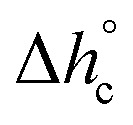
, ±0.02 for *w*_MA_ ± 0.04 for *w*_RA_)

Cooling rate [K min^−1^]	*T*_c_ [°C]	Δ*h*_c_ [J g^−1^]	*X*_C_	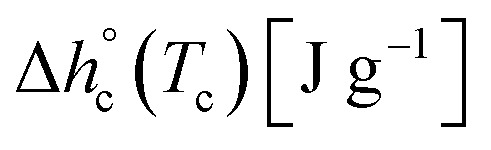	*w*_MA_ (*T*_g_)	*w*_RA_ (*T*_g_)
2	81.8	−75.0	0.41	−183	0.37	0.22
5	76.3	−71.0	0.39	−182	0.37	0.24
10	72.2	−68.2	0.38	−179	0.37	0.25
20	66.5	−65.6	0.38	−173	0.37	0.25

Calculation of PBS crystallinity from the *c*_p,app_ curves shown in Fig. 2 requires an accurate value for the enthalpy of melting of 100% crystalline PBS 
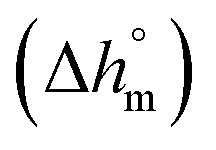
. Unfortunately, largely scattered 
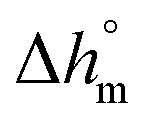
 values are reported in the literature for PBS.^4,18,26,63–66^ The value of 110 J g^−1^, attained with the group contribution method described by van Krevelen,^67^ is often utilized, as well as values included between 200 and 230 J g^−1^.^64–66^ To overcome this problem, the crystalline fraction (*X*_C_) of the PBS samples cooled at different rates was quantified by XRD analysis. Then, the *X*_C_ values were linked to the enthalpy of crystallization (Δ*h*_c_) derived from the *c*_p,app_ curves (Fig. 2a), to attain, in combination with the temperature dependence of the thermodynamic solid and liquid specific heat capacities, the enthalpy of melting of 100% crystalline PBS as a function of temperature.

**Fig. 3:**
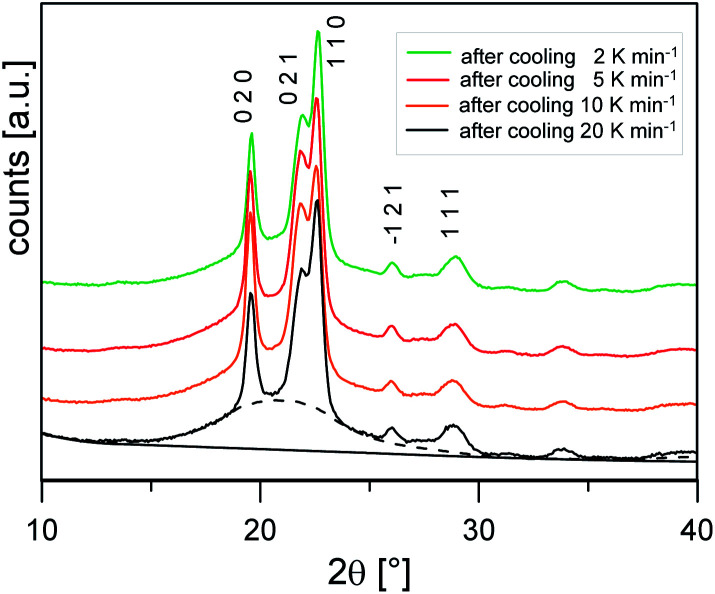
XRD patterns collected at *T*_room_ of the PBS samples cooled at different rates. Miller indexes of the most intense reflections of the PBS α-form are reported. As an example, the scattering of the non-crystalline fraction (dashed lines) and background (solid line) are also shown for the PBS sample cooled at 20 K min^−1^.

Fig. 3 shows the XRD scans at *T*_room_ after cooling at different rates. The profiles show the same set of reflections (the main ones at 2*θ* values: 19.6°, 21.9°, 22.6°, 26.0°, 28.8°), all ascribable to PBS α-form, the modification that commonly grows from the melt.^20^ The bell-shaped baseline, connected to the non-ordered regions of the samples, is roughly maintained with similar intensity, suggesting a limited variation of the samples crystallinity.

**Fig. 4:**
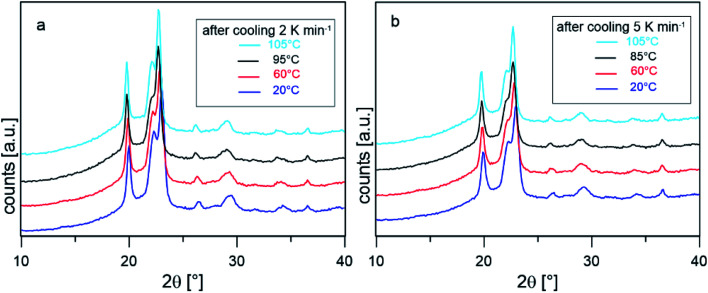
XRD scans collected *in situ* at the indicated temperature after cooling from the melt at 2 K min^−1^ (a) and 5 K min^−1^ (b).

To confirm that the intense reorganization/recrystallization that occurs upon heating does not involve different crystalline structures, XRD analysis was performed on PBS samples after cooling at 2 and 5 K min^−1^, at selected temperatures upon heating at the average rate of 10 K min^−1^. Fig. 4 shows that the XRD profiles registered during heating are all due exclusively to the α-phase. The peak shift towards smaller angles observed as the temperature increases is usual for *in situ* measurements and it is due to thermal expansion of the unit cell, which causes an expansion of interplanar distances. These observations confirm that reorganization/recrystallization occurring upon heating concerns different crystalline populations or morphologies, without change in crystal form. However, as the transformations involve high latent heat exchanges, important modifications of the crystalline organization are expected. This topic will be investigated in detail in a forthcoming study.

The crystalline fractions *X*_C_ of the PBS samples crystallized at different rates are reported in Table 1. The slightly higher crystallinity after lower cooling rate is reflected in a slightly higher *T*_g_ value (*T*_g_ = −33 °C for the sample cooled at 2 K min^−1^, with respect to *T*_g_ = −36 °C for the sample cooled at 20 K min^−1^). From the experimental Δ*h*_c_ values and the crystal fractions *X*_C_, the enthalpies of crystallization of 100% crystalline PBS 
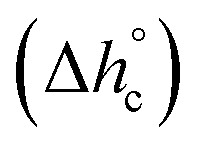
 at *T*_c_ were determined as: 
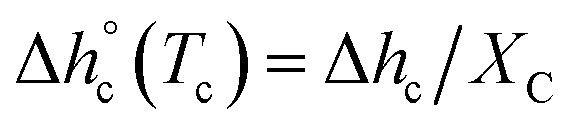
, being negligible the contribution to Δ*h*_c_ of the crystallization at low temperature. Table 1 shows that the absolute value of 
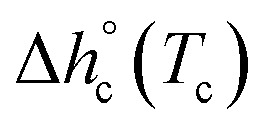
 decreases with decreasing *T*_c_. By taking into account that the enthalpy of melting (Δ*h*_m_) is equal to −Δ*h*_c_, the temperature evolution of the enthalpy of melting of 100% crystalline PBS was determined through the relationship:2
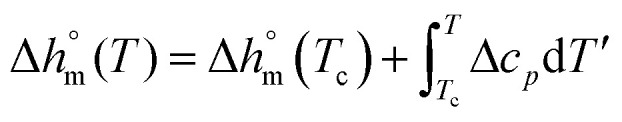
with Δ*c*_p_ = *c*_p,l_ – *c*_p,s_. From eqn (2), the following average expression: 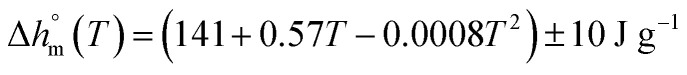 with *T* in °C was obtained. According to this equation, the enthalpy of melting of 100% crystalline PBS at the melting temperature, which is centered at about 113 °C, is 195 ± 10 J g^−1^, which confirms the 
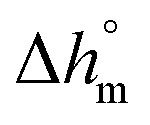
 values reported in the literature close to 200 J g^−1^.^64–66^

From the *c*_p.app_ increment at *T*_g_ (Δ*c*_p,app_), a mobile amorphous weight fraction (*w*_MA_) of 0.37 was calculated for all the samples, as *w*_MA_ = Δ*c*_p,app_/Δ*c*_p,a_. Consequently, the rigid amorphous weight fractions (*w*_RA_) at *T*_g_ were determined by difference, being *X*_C_ + *w*_MA_ + *w*_RA_ = 1 (see Table 1). The trends exhibited by *w*_RA_ and in parallel by *w*_C_ show that the PBS crystals that grow at lower temperatures are coupled with a slightly higher rigid amorphous fraction at *T*_g_.

Rigid amorphous fraction can develop during crystallization, especially at low crystallization temperatures, when the chain mobility is low and the segments arrangements in regular crystalline structures is hindered. But RAF can grow also upon the cooling subsequent to crystallization, due to the progressive reduction in chain mobility and the presence of constraints not completely released in proximity of the crystals. Upon cooling, RAF vitrification occurs in a wide temperature range, depending on the distance from the crystals surfaces and the relative mobility hindrance. The RAF formation is a true vitrification process, because generally mobilization of a RAF portion occurs at the same temperature at which it had previously vitrified upon cooling.^50^

**Fig. 5:**
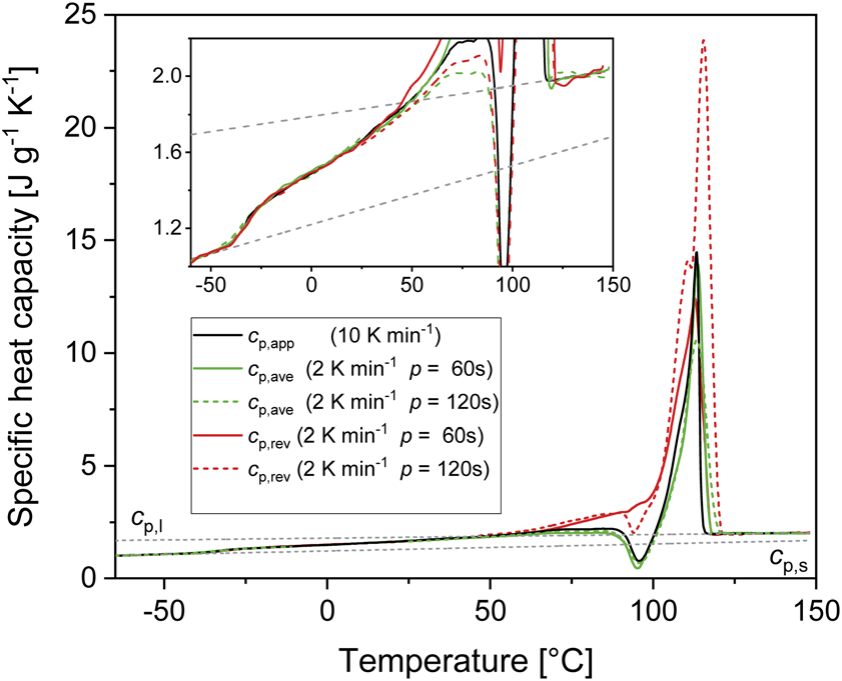
Specific heat capacities of PBS after cooling at 10 K min^−1^ as a function of temperature: apparent specific heat capacity (*c*_p,app_) at 10 K min^−1^, average specific heat capacity (*c*_p,ave_) and reversing specific heat capacity (*c*_p,rev_) at 2 K min^−1^ (*p* = 60 s and *p* = 120 s, *A*_T_ = 0.5 K). The dotted lines are the thermodynamic solid and liquid specific heat capacities (*c*_p,s_ and *c*_p,l_) of PBS. The inset is an enlargement of the specific heat capacity curves.

The RAF evolution of PBS at temperatures higher than *T*_g_ was determined by a comparison of the *c*_p,app_, *c*_p,ave_ and *c*_p,rev_ data after cooling at 10 K min^−1^. Fig. 5 collects the *c*_p,app_ curves at 10 K min^−1^, and the *c*_p,ave_ and *c*_p,rev_ curves at 2 K min^−1^ and *p* = 60 and 120 s. Below *T*_g_ and in the temperature range from *T*_g_ to about 25 °C, the *c*_p,app_ and *c*_p,rev_ curves match within the experimental error. This proves that no reversing latent heat is absorbed or released upon heating from *T*_g_ to about 25 °C, which means that up to about 25 °C the *c*_p,app_ and *c*_p,rev_ correspond to the thermodynamic specific heat capacity of PBS after cooling at 10 K min^−1^. At higher temperatures, the reversing heat capacity becomes dependent on the modulation frequency, attesting the beginning of melting/recrystallization processes. From the thermodynamic specific heat capacity *c*_p,rev_, the mobile amorphous weight fraction (*w*_MA_) was determined as:3
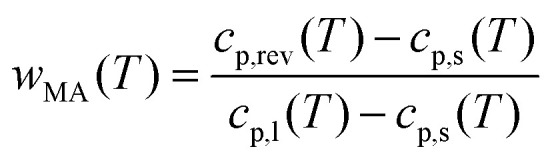


**Fig. 6:**
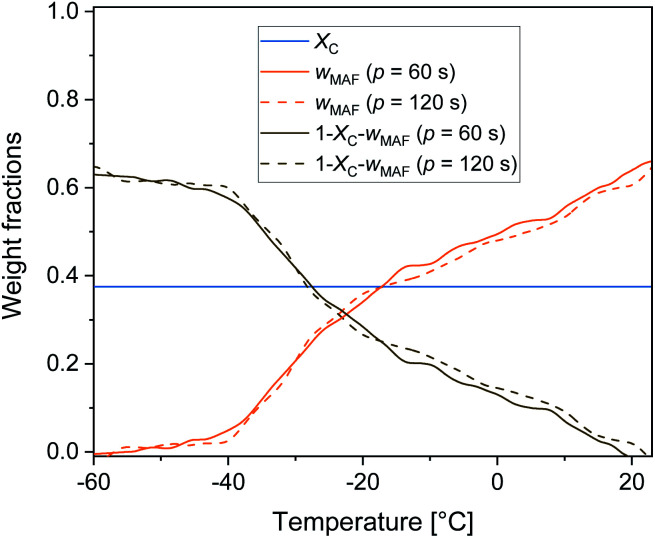
Temperature dependence of crystalline (*X*_C_), mobile amorphous (*w*_MA_) and rigid amorphous (1 − *X*_C_ − *w*_MA_) weight fractions upon heating at 2 K min^−1^ after cooling at 10 K min^−1^. Above *T*_g_, (1 − *X*_C_ − *w*_MA_) corresponds to *w*_RA_, below *T*_g_, (1 − *X*_C_ − *w*_MA_) corresponds to *w*_MA_ + *w*_RA_ (estimated errors: ±0.02 for *X*_C_, ±0.02 for *w*_MA_ ± 0.04 for *w*_RA_).

Fig. 6 displays the temperature evolution of *w*_MA_ and *w*_RA_ up to about 25 °C, with *w*_RA_ calculated by difference: *w*_RA_ = 1 − *X*_C_ − *w*_MA_. Also *X*_C_, which is constant up to about 25 °C is displayed. It is worth noting that below *T*_g_ the mobile amorphous fraction is vitrified, *w*_MA_ = 0, so that (1 − *X*_C_ − *w*_MA_) corresponds to the entire amorphous fractions *w*_MA_ + *w*_RA_. The rigid amorphous weight fraction in PBS is approximately 0.25 at *T*_g_, and decreases with increasing temperature, becoming zero in proximity of 25 °C This means that at *T*_room_ RAF is absent in PBS. This should be true also after different crystallization conditions, because the *c*_p,app_ curves after solidification at different cooling rates were found perfectly overlapping up to about 50 °C (Fig. 2b). As a consequence of the RAF devitrification, the mobile amorphous fraction in parallel increases. At temperatures higher than about 25 °C, melting and recrystallization of imperfect crystals contributes to *c*_p,rev_, reversing latent heat is exchanged and, consequently, correct *w*_MA_ values cannot be obtained through eqn (3). However, the beginning of the melting certainly produces a further MAF increase.

## Conclusions

An accurate thermal characterization of PBS semi-crystalline samples has allowed to quantify the temperature evolution of the rigid amorphous fraction and the temperature dependence of the enthalpy of melting of 100% crystalline PBS. The rigid amorphous weight fraction in PBS is about 0.25 at *T*_g_. It decreases with increasing temperature and becomes zero around 25 °C. Thus, at *T*_room_ and at the human body temperature, in case of biomedical applications, RAF is absent in PBS.

This information is important for a detailed characterization of this biopolymer, by considering the peculiar physical properties of the rigid amorphous fraction, in particular mechanical and permeability properties, which are different from those of the mobile amorphous fraction. It is worth pointing out that an appropriate interpretation of the properties of semi-crystalline polymers as a function of the RAF amount has to be performed by taking into account the true rigid amorphous percentage at the temperature of interest, and not the RAF calculated at *T*_*g*_.

In addition, it is useful to point out that in case of chemical modification of PBS, for example through copolymerization, changes in the amorphous segments mobility are expected, not only in the MAF region, *i.e.* far from the crystal surface, but also in proximity of the crystals, as a consequence of inclusion or, more often, rejection of the co-monomers from the crystal lattice. This could produce different temperature evolution of the RAF, so that the presence of rigid amorphous fraction at *T*_room_ could be tuned by proper co-monomer addition.

## Author contributions

Maria Cristina Righetti: conceptualization, methodology, investigation, formal analysis, writing – original draft, writing – review & editing. Maria Laura Di Lorenzo: formal analysis, writing – review & editing. Patrizia Cinelli: resources, writing – review & editing. Massimo Gazzano: investigation, formal analysis, writing – review & editing.

## Conflicts of interest

There are no conflicts to declare.

## Supplementary Material
